# Association Between Clinically Significant Childhood Trauma and Dissociative Symptoms in a Multicenter Sample of Italian Psychiatric Outpatients

**DOI:** 10.1177/24705470261478704

**Published:** 2026-08-17

**Authors:** Costanzo Frau, Roberta Spiga

**Affiliations:** 1 Department of Psychology, 5289 Manchester Metropolitan University, Manchester, UK; 2 Psychotherapy and Research Centre for Trauma & Dissociation, Cagliari, Italy

**Keywords:** childhood trauma, dissociation, stress, trauma-informed care, psychiatric outpatients, community mental health, childhood maltreatment, epidemiology, mental disorders, Italy

## Abstract

**Background:**

Childhood trauma is highly prevalent among psychiatric populations and represents a major risk factor for a wide range of mental disorders. Exposure to childhood maltreatment has also been consistently associated with dissociative symptomatology, yet epidemiological data from Italian psychiatric outpatient populations remain limited. This study examined the prevalence of clinically significant childhood trauma and its association with dissociative symptoms in a multicentre sample of Italian psychiatric outpatients.

**Methods:**

This cross-sectional study included 104 psychiatric outpatients recruited from Community Mental Health Centres across Sardinia. Participants completed the Childhood Trauma Questionnaire–Short Form (CTQ-SF) and the Dissociative Experiences Scale-II (DES-II). Participants scoring at or above the moderate threshold on at least one CTQ-SF subscale were classified as trauma-exposed. Descriptive analyses examined the prevalence of clinically significant childhood trauma exposure, and group comparisons were conducted to evaluate differences in dissociative symptoms.

**Results:**

Clinically significant childhood trauma exposure was reported by 76.9% of participants. Trauma-exposed participants reported significantly higher levels of dissociative symptoms than non-trauma-exposed participants (M = 24.3 vs. 16.1). An independent-samples t-test indicated a significant group difference, t(102) = 1.99, p = .049, with a moderate effect size (d = 0.46).

**Conclusions:**

Childhood trauma and dissociative symptoms appear highly prevalent among individuals receiving care in community mental health services. These findings support the clinical relevance of routinely assessing both trauma exposure and dissociative symptomatology and highlight the potential value of trauma-informed approaches within public mental health settings.

## 1. Introduction

Adverse childhood experiences (ACEs) and cumulative interpersonal trauma are increasingly recognized as major public mental health concerns that contribute substantially to the development and persistence of psychiatric disorders across the lifespan.^1-4^

Large epidemiological investigations conducted within the National Comorbidity Survey Replication and the World Mental Health Surveys have consistently shown that childhood adversities are highly prevalent and strongly associated with mood, anxiety, psychotic, substance use, and personality disorders across different stages of life.^1,2^ Furthermore, recent epidemiological and meta-analytic evidence suggests that childhood maltreatment contributes substantially to the overall burden of mental disorders, suicidality, disability, and healthcare utilization.^4,5^

Beyond its established association with psychiatric morbidity, cumulative trauma exposure has been increasingly linked to dissociative symptomatology. Dissociation refers to disruptions in the integration of consciousness, memory, identity, emotion, perception, and bodily experience, ranging from relatively common dissociative experiences to more severe pathological manifestations.^6^ Although dissociative symptoms have traditionally been associated with dissociative disorders and trauma-related conditions, growing evidence indicates that dissociation represents a clinically relevant transdiagnostic phenomenon observable across multiple psychiatric disorders, including depression, anxiety disorders, psychosis, personality disorders, eating disorders, and somatic symptom disorders.^6-9^ Recent findings further suggest that dissociation may constitute an underrecognized contributor to cumulative psychiatric burden, functional impairment, and poorer treatment outcomes within mental health populations.^10,11^

Trauma-related dissociation appears particularly relevant in the context of developmental and relational trauma. The traumatic-dissociative dimension framework proposes that repeated interpersonal trauma and attachment-related adversities may contribute to dissociative pathogenic processes that complicate clinical presentations across diagnostic categories.^12^ Similarly, recent theoretical and clinical contributions have emphasized the pervasive impact of childhood relational trauma and dissociative symptoms across the lifespan, highlighting their association with emotional dysregulation, chronic psychopathology, and increased clinical complexity.^13^ Despite these findings, dissociative symptoms often remain insufficiently assessed within routine psychiatric care, particularly in public and community mental health settings.

A substantial body of research has documented a robust association between cumulative trauma exposure and dissociative symptomatology across both clinical and community populations. Meta-analytic evidence suggests that childhood maltreatment, particularly chronic interpersonal and attachment-related trauma, is consistently associated with elevated levels of psychoform and somatoform dissociation.^6^ Consistent findings have also been reported across different psychiatric populations, including patients with schizophrenia, panic disorder, and depressive or anxiety disorders, where childhood trauma has been associated with greater dissociative symptom severity and increased clinical complexity.^9,14,15^ These findings support the hypothesis that dissociation may represent a clinically relevant mechanism linking developmental trauma to a broad range of psychiatric manifestations. Moreover, much of the available evidence has been derived from highly selected clinical populations or meta-analytic syntheses.^4,6^ Consequently, relatively little is known about the prevalence and clinical correlates of dissociative symptoms among diagnostically heterogeneous psychiatric outpatients receiving routine care in public community mental health services.

While international research has consistently demonstrated the association between childhood trauma and dissociative symptoms across psychiatric populations, epidemiological evidence from Italian psychiatric outpatient settings remains limited. In particular, few multicentre studies have investigated these associations within public community mental health services using clinically validated measures of both childhood trauma and dissociative symptoms. Addressing this gap may improve the identification of trauma-related clinical complexity and support the implementation of trauma-informed psychiatric care in routine psychiatric practice.

The present study aimed to examine the prevalence of clinically significant childhood trauma and its association with dissociative symptoms in a multicenter sample of Italian psychiatric outpatients. Specifically, we investigated whether participants with clinically significant childhood trauma exposure reported higher levels of dissociative symptomatology than those without trauma exposure.

## 2. Methods

### 2.1. Study Design

This cross-sectional observational study was conducted as part of an ongoing multicenter regional epidemiological investigation of childhood trauma and dissociative symptoms among psychiatric outpatients in Sardinia, Italy. The study aimed to estimate the prevalence of clinically significant childhood trauma and examine its association with dissociative symptom severity in a heterogeneous psychiatric outpatient population.

### 2.2. Setting and Participants

Participants were recruited from multiple Community Mental Health Centers across Sardinia, Italy. Eligible participants were adults aged 18–65 years receiving outpatient psychiatric treatment. Exclusion criteria included severe cognitive impairment, intellectual disability, or any condition preventing completion of the study questionnaires. A total of 104 participants were enrolled. Descriptive analyses were conducted using all available data. Group comparisons examining the association between childhood trauma and dissociative symptoms were performed on participants with complete CTQ-SF and DES-II data.

### 2.3. Ethics

The study was conducted in accordance with the ethical principles outlined in the Declaration of Helsinki and was approved by the Ethics Committee of [institution masked for review]. Written informed consent was obtained from all participants prior to study participation. Participation was voluntary, and all data were collected and analyzed anonymously.

### 2.4. Measures

#### 2.4.1. Clinical and Sociodemographic Variables

Sociodemographic and clinical information was obtained through structured assessment and clinical records, including age, sex, psychiatric diagnosis, and other relevant clinical characteristics.

#### 2.4.2. Childhood Trauma

Childhood traumatic experiences were assessed using the Childhood Trauma Questionnaire–Short Form (CTQ-SF),^16,17^ a widely used 28-item self-report instrument designed to assess five dimensions of childhood maltreatment: emotional abuse, physical abuse, sexual abuse, emotional neglect, and physical neglect. Items are rated on a five-point Likert scale ranging from 1 (“Never True”) to 5 (“Very Often True”), with higher scores indicating greater exposure to childhood adversity. The CTQ-SF has demonstrated good reliability and validity across both clinical and non-clinical populations and is one of the most widely used measures of retrospective childhood maltreatment.^16^

#### 2.4.3. Dissociative Symptoms

Dissociative symptoms were assessed using the Italian version of the Dissociative Experiences Scale-II (DES-II),^18,19^ a widely used 28-item self-report measure designed to assess the frequency of dissociative experiences in everyday life. Participants indicate the percentage of time they experience each dissociative phenomenon on a scale ranging from 0% to 100%, with higher scores reflecting greater dissociative symptomatology. The DES-II has demonstrated good reliability and validity across both clinical and non-clinical populations.^18,20,21^ The Italian version has shown satisfactory psychometric properties, including good internal consistency, test–retest reliability, and convergent validity in both clinical and community samples.^19^ However, to our knowledge, no validated diagnostic cut-off with corresponding sensitivity and specificity estimates has been established for the Italian version of the DES-II. Accordingly, the DES-II total score was analyzed as a continuous measure of dissociative symptom severity.

The DES-II total score was used as the primary measure of dissociative symptom severity.

### 2.5. Statistical Analyses

Descriptive statistics were used to summarize participants’ sociodemographic and clinical characteristics and to estimate the prevalence of clinically significant childhood trauma. Continuous variables are presented as means and standard deviations (SDs), whereas categorical variables are reported as frequencies and percentages.

Participants were classified as trauma-exposed if they scored at or above the moderate threshold on at least one CTQ-SF subscale. Between-group differences in dissociative symptom severity (DES-II total score) were examined using independent-samples t-tests, with effect sizes estimated using Cohen’s *d*. Because DES-II scores were not normally distributed, a Kruskal–Wallis test was additionally performed to assess the robustness of the findings. Statistical significance was set at *p* < .05. All analyses were conducted using JAMOVI (Version 2.6).

## 3. Results

### 3.1. Sample Characteristics

Table 1 summarizes the sociodemographic and clinical characteristics of the sample. Participants had a mean age of 39.4 years (SD = 15.2) and a mean educational level of 11.2 years (SD = 3.13). The majority of participants were female (69.9%). Mood disorders were the most common diagnostic category, followed by anxiety and trauma-related disorders and personality disorders. The sociodemographic and clinical characteristics of the sample are presented in Table 1.

**Table 1.** Sociodemographic and Clinical Characteristics of the Sample (N = 104)

Characteristic	Value
Age, years, mean (SD)	39.4 (15.2)
Years of education, mean (SD)	11.2 (3.13)
**Sex, n (%)**	
Female	72 (69.9)
Male	29 (28.2)
Missing	2 (1.9)
**Primary psychiatric diagnosis, n (%)**	
Mood Disorders	26 (25.0)
Anxiety and Trauma-Related Disorders	22 (21.2)
Personality Disorders	16 (15.4)
Adjustment Disorders	9 (8.7)
Psychotic Disorders	8 (7.7)
Other Disorders	5 (4.8)
Substance Use Disorders	1 (1.0)

*Note.* Continuous variables are presented as mean (SD). Categorical variables are presented as frequencies and percentages. Psychiatric diagnosis was unavailable for 17 participants.

### 3.2. Prevalence of Trauma

Among the 104 participants with available CTQ-SF data, 80 (76.9%) met criteria for clinically significant childhood trauma exposure, whereas 24 (23.1%) did not. Clinically significant trauma exposure was defined as scoring at or above the moderate threshold on at least one CTQ-SF subscale.

These findings indicate a high prevalence of childhood trauma among Italian psychiatric outpatients. More than three-quarters of participants reported at least one form of clinically significant childhood maltreatment, suggesting that trauma-related experiences represent a common feature of individuals accessing public mental health services.

Overall, the results support previous epidemiological findings indicating that childhood adversities are highly prevalent among individuals with psychiatric disorders and may contribute substantially to clinical complexity within community mental health settings.

### 3.3. Dissociation Findings

To examine the association between childhood trauma exposure and dissociative symptomatology, participants were divided into trauma-exposed and non-trauma-exposed groups according to CTQ-SF criteria. Participants with clinically significant trauma exposure reported significantly higher levels of dissociative symptoms on the Dissociative Experiences Scale-II (DES-II) than participants without trauma exposure. As shown in Table 2, the trauma-exposed group obtained a mean DES-II score of 24.3 (SD = 18.4), whereas the non-trauma-exposed group obtained a mean score of 16.1 (SD = 15.1).

**Table 2.** Comparison of Dissociative Symptoms Between Trauma-Exposed and Non-trauma-exposed Participants

Group	N	DES-II mean (SD)
No trauma exposure	24	16.1 (15.1)
Trauma exposure	80	24.3 (18.4)

*Note.* Trauma exposure was defined as scoring at or above the moderate threshold on at least one CTQ-SF subscale.

An independent-samples t-test revealed a statistically significant difference between groups, t(102) = 1.99, p = .049, with a moderate effect size (Cohen’s d = 0.46). To account for the non-normal distribution of DES-II scores, a non-parametric Kruskal–Wallis test was also performed. The result confirmed the association between trauma exposure and dissociative symptoms, χ^2^(1) = 5.10, p = .024.

To examine whether the observed association remained after adjustment for sex, an analysis of covariance (ANCOVA) was performed. The association between childhood trauma and dissociative symptoms remained statistically significant after adjustment, *F*(1,100) = 4.93, *p* = .029.

For descriptive purposes, we also examined the proportion of participants scoring above the conventional DES-II threshold of ≥30, which has frequently been used in the literature as an indicator of elevated dissociative symptomatology. Twenty-eight of the 104 participants (26.9%) scored above this threshold.

Overall, participants reporting clinically significant childhood trauma exposure showed substantially higher levels of dissociative symptomatology than participants without trauma exposure, supporting the hypothesis that childhood trauma is associated with increased dissociative experiences in community psychiatric populations.

## 4. Discussion

### 4.1. Main Findings

The present study investigated the prevalence of clinically significant childhood trauma and its association with dissociative symptoms in a multicenter sample of Italian psychiatric outpatients. Three main findings emerged.

First, clinically significant childhood trauma was highly prevalent, with more than three-quarters of participants reporting exposure to at least one form of childhood maltreatment. This finding indicates that adverse developmental experiences are common among psychiatric outpatients and reinforces evidence identifying childhood trauma as a major contributor to adult psychopathology.

Second, participants with clinically significant childhood trauma reported significantly higher levels of dissociative symptoms than those without trauma exposure. This association was confirmed by both parametric and non-parametric analyses, supporting the robustness of the relationship between childhood trauma and dissociative symptom severity.

Third, the findings highlight the clinical relevance of dissociative symptoms across diagnostically heterogeneous psychiatric populations. The observed association suggests that dissociation may represent an important but frequently underrecognized dimension of psychopathology that deserves greater attention during routine psychiatric assessment.

### 4.2. Comparison With Previous Literature

The present findings are broadly consistent with international epidemiological research demonstrating strong associations between childhood adversity and adult mental disorders. Large-scale investigations, including the National Comorbidity Survey Replication and the WHO World Mental Health Surveys, have shown that childhood adversities are highly prevalent and associated with increased risk for a wide range of psychiatric conditions across the lifespan.^1,2^ Similarly, longitudinal and meta-analytic evidence has confirmed childhood trauma as a significant risk factor for subsequent psychopathology.^4^

The observed association between trauma exposure and dissociative symptoms is also consistent with contemporary trauma and dissociation literature. Developmental and attachment-related trauma has repeatedly been linked to dissociative phenomena across diagnostic categories, supporting the notion of a transdiagnostic traumatic-dissociative dimension.^12,13^ Furthermore, meta-analytic evidence indicates that dissociative symptoms are not limited to dissociative disorders but occur across a broad spectrum of psychiatric conditions, including mood, anxiety, personality, psychotic, and substance-related disorders.^6^

Although dissociation has traditionally been assessed using global measures such as the DES-II, contemporary conceptualizations increasingly regard dissociation as a multidimensional construct comprising partially distinct domains.^22-24^ Recent synthesis work further indicates that, despite this conceptual shift, most empirical studies continue to rely on DES-II total scores rather than domain-specific analyses.^24^ Accordingly, the present findings should be interpreted as reflecting an association between childhood trauma and overall dissociative symptom severity, rather than specific dissociative domains, an issue that warrants further investigation in future studies.

Although the present findings are broadly consistent with previous international evidence, they provide preliminary epidemiological data from Italian public outpatient mental health services, a clinical setting that remains substantially underrepresented in trauma and dissociation research. By examining a diagnostically heterogeneous sample of psychiatric outpatients receiving routine care, the present study extends existing epidemiological evidence and provides clinically relevant information on the prevalence of childhood trauma and its association with dissociative symptom severity in everyday psychiatric practice.

To our knowledge, this is among the first multicentre studies conducted in Italian public community mental health services to examine the association between clinically significant childhood trauma and dissociative symptoms across a diagnostically heterogeneous psychiatric outpatient population.

Importantly, the present study represents the first phase of an ongoing multicentre epidemiological project investigating childhood trauma and dissociative symptoms among psychiatric outpatients. The analyses reported here are based on the initial study sample and should therefore be considered preliminary. As participant recruitment is ongoing, completion of the project will enable investigation of dose-response relationships, multidimensional conceptualizations of dissociation, domain-specific associations between childhood maltreatment and dissociative symptoms, emotion regulation processes, and potential differences across diagnostic groups. These future analyses are expected to provide a more comprehensive understanding of trauma-related psychopathology within routine community mental health care.

### 4.3. Clinical Implications

The present findings have several clinical implications. First, the high prevalence of childhood trauma observed in this sample reinforces the importance of routinely considering developmental trauma during psychiatric assessment and treatment planning. Childhood maltreatment may substantially contribute to the clinical presentation of psychiatric disorders, even when trauma-related symptoms are not the primary reason for referral.

Second, the observed association between childhood trauma and dissociative symptoms highlights the importance of assessing dissociation as part of routine psychiatric evaluation. Dissociative symptoms may contribute to emotional dysregulation, functional impairment, and overall clinical complexity, yet they frequently remain underrecognized in everyday psychiatric practice.^6,12^ Failure to identify dissociative phenomena may result in incomplete case formulation and less effective treatment planning.

These findings support the integration of trauma-informed principles into routine psychiatric care. The use of brief, validated screening instruments for childhood trauma and dissociation may facilitate earlier identification of clinically vulnerable individuals and improve diagnostic formulation. Greater awareness of trauma-related and dissociative psychopathology may also support more individualized treatment planning and ultimately contribute to improved clinical outcomes across heterogeneous psychiatric populations.

### 4.4. Limitations and Strengths

Several limitations should be considered when interpreting the present findings. First, the cross-sectional design precludes any causal inference regarding the relationship between childhood trauma and dissociative symptoms. Although trauma exposure was associated with higher levels of dissociative symptoms, the temporal and causal mechanisms underlying this association cannot be established.

Second, the study was conducted in a regional community psychiatric sample, which may limit the generalizability of the findings to other mental health settings or populations. Replication in larger and more diverse samples is therefore warranted.

Third, the relatively small and diagnostically heterogeneous sample precluded investigation of the multidimensional structure of dissociation, domain-specific dissociative symptoms, or differential associations between specific forms of childhood maltreatment and distinct dissociative dimensions. Accordingly, the present findings should be interpreted as reflecting overall dissociative symptom severity rather than specific dissociative domains. Future studies with larger samples should examine these associations using multidimensional models of dissociation.

Similarly, the use of a binary classification of childhood trauma based on established CTQ-SF clinical cut-offs did not allow examination of dose-response relationships or differential associations between specific maltreatment subtypes and distinct dissociative domains. Future studies using larger and diagnostically more homogeneous samples should investigate these associations using continuous measures of childhood trauma and multidimensional conceptualizations of dissociation.

Finally, childhood trauma and dissociative symptoms were assessed using self-report measures. Although both the CTQ-SF and DES-II are widely used, psychometrically validated instruments, retrospective reporting may be influenced by recall bias and subjective interpretation. However, recent methodological work has questioned the assumption that clinician-administered interviews should invariably be regarded as the gold standard for assessing subjective psychological experiences, highlighting that both self-report and interview-based assessments are subject to different sources of measurement error.^25^ Future studies integrating multiple assessment approaches may provide a more comprehensive evaluation of childhood trauma and dissociative symptoms.

In addition, although the DES-II is among the most widely used and psychometrically validated instruments for screening dissociative symptoms, its primary purpose is the identification of clinically relevant dissociative experiences rather than a comprehensive assessment of the full spectrum of dissociative psychopathology. Future research may therefore benefit from incorporating more comprehensive multidimensional measures or structured diagnostic interviews, such as the Multidimensional Inventory of Dissociation (MID), the Structured Clinical Interview for DSM Dissociative Disorders (SCID-D), or the Dissociative Disorders Interview Schedule (DDIS), particularly when the objective is to characterize specific dissociative symptom profiles or establish formal dissociative diagnoses.

Despite these limitations, the study has several strengths. It was conducted in a real-world community mental health setting and included a diagnostically heterogeneous psychiatric sample representative of routine clinical practice. Furthermore, the study provides preliminary epidemiological evidence from Italian public outpatient mental health services, a clinical setting that remains substantially underrepresented in trauma and dissociation research. By focusing on clinically relevant phenomena that are frequently overlooked during routine psychiatric assessment, the findings contribute to the growing literature supporting trauma-informed approaches within community mental health services.

## 5. Conclusions

The present study provides further evidence that clinically significant childhood trauma is highly prevalent among psychiatric outpatients and is associated with greater dissociative symptom severity. These findings support the growing recognition of childhood trauma and dissociation as clinically relevant transdiagnostic dimensions of psychopathology. Routine assessment of trauma exposure and dissociative symptoms may improve diagnostic formulation, facilitate more individualized treatment planning, and contribute to the delivery of trauma-informed psychiatric care. Future multicenter studies involving larger and more diverse psychiatric populations are warranted to further clarify the clinical implications of these findings.

## Data Availability

The datasets generated and/or analyzed during the current study are available from the corresponding author upon reasonable request.*
